# Multi-Modal Portable Respiratory Rate Monitoring Device for Childhood Pneumonia Detection

**DOI:** 10.3390/mi14040708

**Published:** 2023-03-23

**Authors:** Sadeque Reza Khan, Xiaohan Wang, Tiantao Jiang, Wei Ju, Norbert Radacsi, Muhammad Abdul Kadir, Khondkar Siddique-e Rabbani, Steve Cunningham, Srinjoy Mitra

**Affiliations:** 1 School of Engineering and Physical Sciences, Institute of Sensors, Signals and Systems, Heriot-Watt University, Edinburgh EH14 4AS, UK; 2 School of Engineering, Institute of Integrated Micro and Nano Systems, The University of Edinburgh, Edinburgh EH9 3FF, UK; 3 School of Engineering, Institute for Materials and Processes, The University of Edinburgh, Robert Stevenson Road, Edinburgh EH9 3FB, UK; 4 Department of Biomedical Physics and Technology, University of Dhaka, Dhaka 1000, Bangladesh; 5 Centre for Inflammation Research, The University of Edinburgh, Edinburgh EH9 3FF, UK

**Keywords:** accelerometer, bioimpedance, breath per minute, low- and middle-income countries, respiratory rate, textile electrode

## Abstract

Accurate assessment of Respiratory Rate (RR) is the most important mechanism in detecting pneumonia in low-resource settings. Pneumonia is a disease with one of the highest mortality rates among young children under five. However, the diagnosis of pneumonia for infants remains challenging, especially in low- and middle-income countries (LMIC). In such situations, RR is most often measured manually with visual inspection. Accurate RR measurement requires the child to remain calm without any stress for a few minutes. The difficulty in achieving this with a sick child in a clinical environment can result in errors and misdiagnosis, even more so when the child is crying and non-cooperating around unfamiliar adults. Therefore, we propose an automated novel RR monitoring device built with textile glove and dry electrodes which can make use of the relaxed posture when the child is resting on the carer’s lap. This portable system is non-invasive and made with affordable instrumentation integrated on customized textile glove. The glove has multi-modal automated RR detection mechanism that simultaneously uses bio-impedance and accelerometer data. This novel textile glove with dry electrodes can easily be worn by a parent/carer and is washable. The real-time display on a mobile app shows the raw data and the RR value, allowing a healthcare professional to monitor the results from afar. The prototype device has been tested on 10 volunteers with age variation of 3 years to 33 years, including male and female. The maximum variation of measured RR with the proposed system is ±2 compared to the traditional manual counting method. It does not create any discomfort for either the child or the carer and can be used up to 60 to 70 sessions/day before recharging.

## 1. Introduction

Pneumonia is a lung disease caused by a bacterial or viral infection. It is the cause of death for over 1.4 million children under the age of five every year around the world [1]. In 2019, pneumonia accounted for 14% of global child deaths [2]. Although many childhood pneumonia deaths could be prevented with simple early interventions and appropriate treatment, pneumonia often goes undiagnosed and untreated in low-income communities until the child is severely ill. In South-Asian and Sub-Saharan low-resource settings, community diagnosis of pneumonia is based on subjective clinical signs and symptoms given by the World Health Organization iCCM guidelines [3]. This includes cough and fast respiratory rate (RR). Additional features, i.e., chest indrawing, stridor, adventitious sounds on auscultation, or low pulse oxygen saturation, support a diagnosis of severe pneumonia. Despite fast breathing being one of the key diagnostic signs for early pneumonia, in practice, RR measurement techniques for children carry a critical margin of error [4].

RR is usually defined as the number of breaths per minute (or BPM) and is affected by various physical and psychological factors. In low- and middle-income countries (LMIC), counting the number of breaths typically is performed manually with the aid of watches, timers, and counting beads [5]. Measuring a sick child’s RR through visual observation requires focused concentration and can be challenging for a child who may be moving, crying, or breathing rapidly. These problems get exacerbated in crowded clinical settings in low-income countries where the community health workers or clinicians are busy, and the child may be uncooperative or unsettled. Pneumonia can be also diagnosed by lung X-rays, blood tests, or calculating RR from Electrocardiography (ECG) and Photoplethysmography (PPG). None of these is easily available in rural healthcare settings in South Asia or sub-Saharan Arica (where mortality rates are the highest [6]).

RR is one of the few vital signs that still rely on skilled clinical observation and not electronic confirmation. The guidelines of Integrated Management of Childhood Illness and iCCM define 60 or more breaths per minute in infants younger than 2 months, 50 or more breaths per minute in infants aged 2 to 11 months, and 40 or more breaths per minute in children aged 12 to 59 months as the fast breathing [7]. RR can be measured directly by detecting changes in lung volume and body movement using sensors including accelerometers, gyroscopes, pressure sensors, electromagnetic sensors, radar, and WIFI. However, there are limited devices designed specifically to determine RR for childhood pneumonia. A recently launched instrument developed by Philips (ChARM: Children’s Automated Respiration Monitor) specifically targets this demography [8]. However, this accelerometer-based device requires a belt to be applied around the patient, something which may disturb a sick child and thus change their respiratory rate as a result of the test application [9]. RespiraSense is another wearable solution to measure RR. It uses a piezoelectric array of pressure sensors and converts the deformations in the relative angles of the thoracic and abdominal surfaces that occur during breathing to electric signals [10]. The data can be sent by using Wi-Fi or Bluetooth. However, this system is currently too bulky and uncomfortable for an infant to wear. Other commonly used techniques to detect RR are electrical impedance pneumography (EIP), electrical impedance tomography (EIT), and respiratory inductance plethysmography (RIP) [11]. EIP and EIT are both focused on the bio-impedance measurement of the lung. However, using such chest-attachment equipment on an infant or a young child can likely cause discomfort, leading to distress, crying, and uncontrolled movements, which are the main reasons for inaccurate RR measurements [12]. Common to many RR counters currently being evaluated is the degree of training required for use in community settings, which may limit their fidelity [7]. Furthermore, it is still unclear whether bio-impedance or acceleration can provide the most accurate RR data for children [13]. In previous studies [14,15,16], a non-contact RR monitoring system demonstrated promising results using a low-cost RGB camera. However, this method still requires a cooperating child in the correct position to the camera without breaking into sudden movements. One of the most common methods to measure RR is to use contact-based electrodes to detect the variation in bio-impedance caused during respiration. Our proposed device is primarily based on this method; hence, in the following sub-section, we review some electrode materials that could be used for this purpose.

### Evaluation of Electrode Material

There are various potential electrodes available for such an application. Gel electrode is one of the most common types of electrodes used clinically. Gel electrodes can provide better signal integrity with lower noise. However, it can cause skin irritation including redness and itching for long-term use [17]. Furthermore, gel electrodes can only be used once for a single patient. Electrodes made of tiny needles can have the lowest contact impedance and evaluate corresponding bio-signals more precisely. The length of the microneedle electrodes varies based on the application. In [18], a microneedle electrode of 550 µm length fabricated on non-toxic polyimide film with a thickness of 0.3 mm for electromyography (EMG) and ECG recording is presented. These electrodes are less affected by noise and interference. However, these electrodes can cause minor bleeding and inflammation during measurement. Dry electrodes can be made of any conductive materials that can contact the skin. Compared with a gel electrode, the contact impedance between the dry electrode and the skin will be larger and the dry electrode itself is not sticky and needs to be fixed in the proper locations on the skin by external means. The contact impedance of dry electrodes has been studied for a long time [19], including the influence of sweat [20]. While metal dry electrodes can measure accurate bio-potential signals without being affected by motion artifacts [21], they do not ensure user comfort. Furthermore, for our application, the touch of cold metal on the skin of a child can create unease. Compared to a metal electrode, a foam-based dry electrode is more suitable for the uneven skin surface. The stiffness of metal electrodes can cause a micron-wide air gap, which has a huge impact on the contact resistance and motion artifact [22].

With the rising popularity of wearable medical devices, textile electrodes have become a popular field of research. The advantages of this electrode are its flexibility, washability, comfort, and ability to integrate into daily clothing. Textile electrodes are generally manufactured by integrating conductive materials on cloth by weaving, knitting, or electrochemical processing (such as electroplating, dip coating, and printing) [23]. The conductive materials used in the textile electrodes can be metal, conductive polymer, graphene, and carbon nanotube [24]. Carbon electrodes are also a popular new type of dry electrode which is very soft and biocompatible. Application-specific carbon electrodes can be produced by adding different chemical materials to carbon composite [25]. However, carbon electrodes require a complicated manufacturing process which is not yet convenient for mass production. Their long-term mechanical stability is also a matter of concern. Table 1 summarizes the performance of different electrodes mentioned in this section based on electrode–tissue impedance (ETI), level of user comfort, preparation cost, and any major issues.

**Figure 1:**
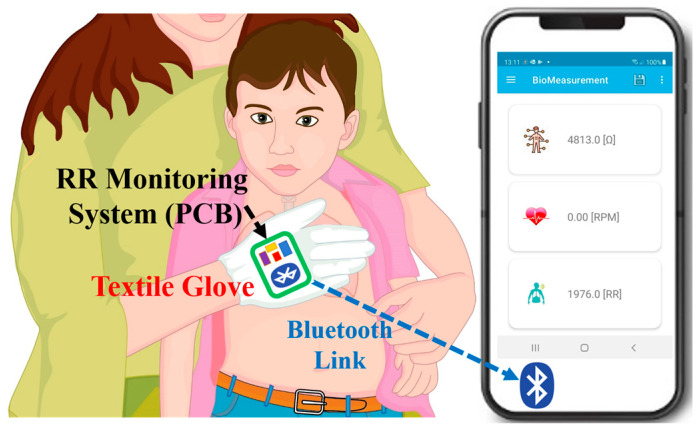
Proposed novel RR monitoring system for infants based on textile glove and dry electrodes.

**Figure 2:**
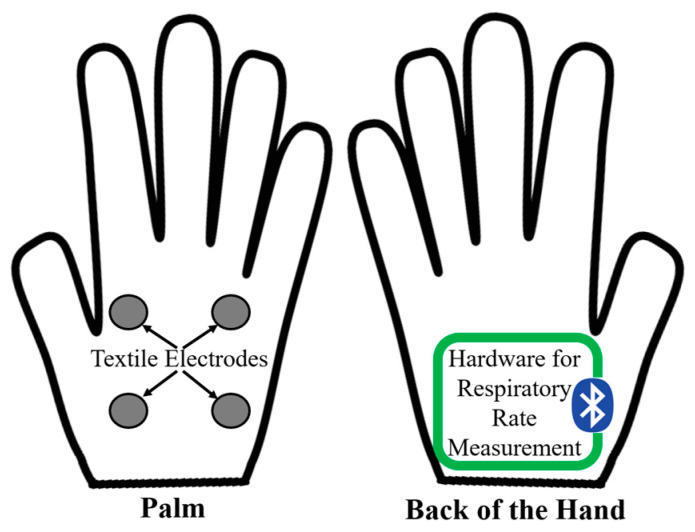
Position of textile electrodes and hardware system in proposed textile glove for RR monitoring.

In this work, we propose a novel multi-modal RR monitoring device specifically designed for infants and children under five in LMICs (Figure 1). The proposed system can measure the RR through well-known bio-impedance measurement and accelerometer data. We intend to create a device that can be easily used by the carer with whom the child is comfortable. This novel device is based on an instrumented glove that can be worn by the adult and lightly placed on the child’s chest. An early version of a similar device has been shown to be effective in a test setup in Bangladesh [26]. In order to reduce any additional distress while using the glove, the material of the electrode should be comfortable on a child’s skin. In Figure 2, the front side of the glove is shown with the position of the textile electrodes. The hardware of the system built on a small PCB is included on the backside of the glove. The processed data can be sent to a mobile phone using Bluetooth.

## 2. Dry Electrode Manufacturing and Characterization

The key criteria to select and manufacture an electrode for the proposed system are the comfort and safety of infants and young children. Cost is another important factor as one of the primary target users is LMICs. Therefore, conductive textile electrodes and flexible carbon electrodes are the two relevant candidates because of their improved electrode-skin contact impedance, softness, and cost, as described in Table 1.

**Figure 3:**
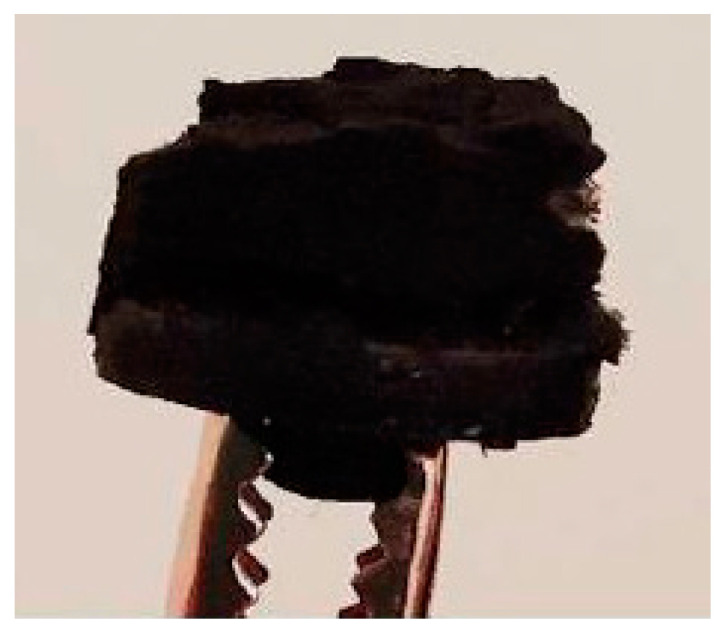
Manufactured soft carbon electrode.

Flexible soft carbon electrodes were manufactured using polypropylene cyanide polymer by utilizing electrospinning and carbonization processes [27]. One of the major issues with carbon electrodes was the limited control over their shape during the manufacturing process. This was due to the random shape of the fiber aggregation during the electrospinning process and the shape of the sticky fiber when it was put into the carbonization furnace, as shown in Figure 3. The performance of the manufactured electrode was measured using electrical impedance spectroscopy.

Friction can easily damage soft carbon electrodes during use. To reduce potential damage and extend service life, electrodes are commonly wrapped in material, which also increases user comfort. However, this complex manufacturing process, limited durability, and the associated cost make soft carbon electrodes a less favorable candidate for this work. Therefore, dry textile electrodes are considered the primary choice for this work.

**Figure 4:**
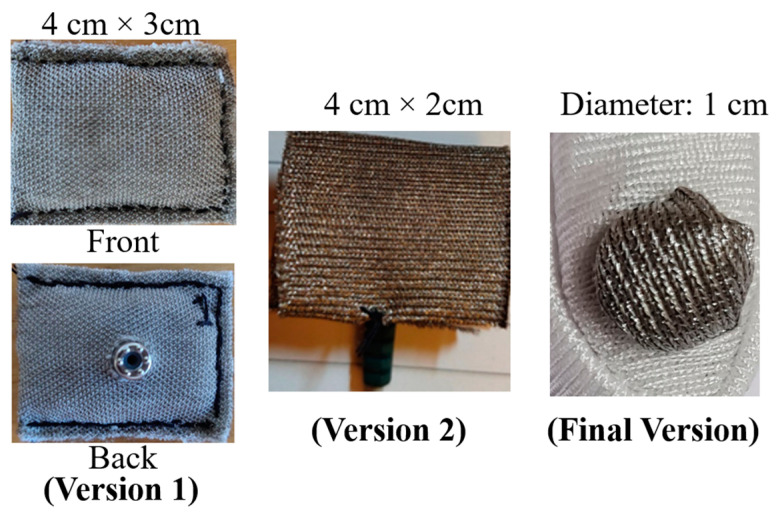
Investigation of textile electrodes of different shape and size.

**Figure 5:**
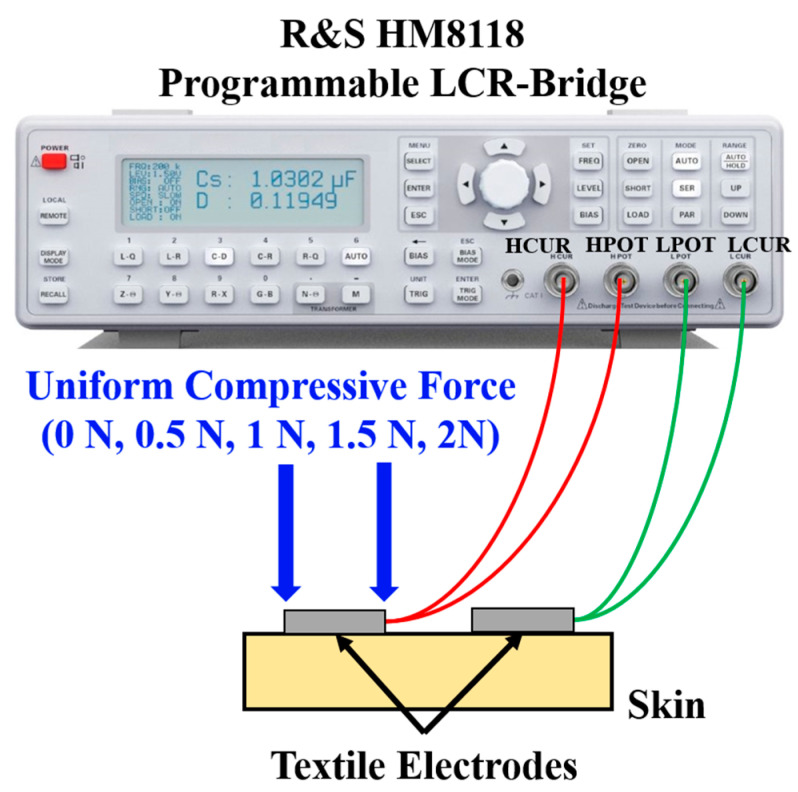
ETI Measurement using LCR bridge.

Two different materials made of silver-plated nylon and cotton (63%), silver yarn (35%), and spandex (2%) with resistance of 1 Ω/foot and 460 Ω/foot were considered. The silver-plated nylon felt smoother and had better stretchability, which made it ideal for our application. A soft filler material was needed to manufacture the electrodes using textile as it was sensitive to high pressure. In Figure 4, textile electrodes of different shapes and sizes were investigated to decide the final architecture. In version 1 (V1), a pillow-shaped textile electrode of size 4 cm × 3 cm with cotton as a filler material was manufactured. The performance of the electrode was evaluated using ETI measurement [28]. ETI measurement was carried out with an R&S HM8118 programmable LCR meter with a frequency range of 0.1 Hz to 200 kHz with 35 test points, as shown in Figure 5.

**Figure 6:**
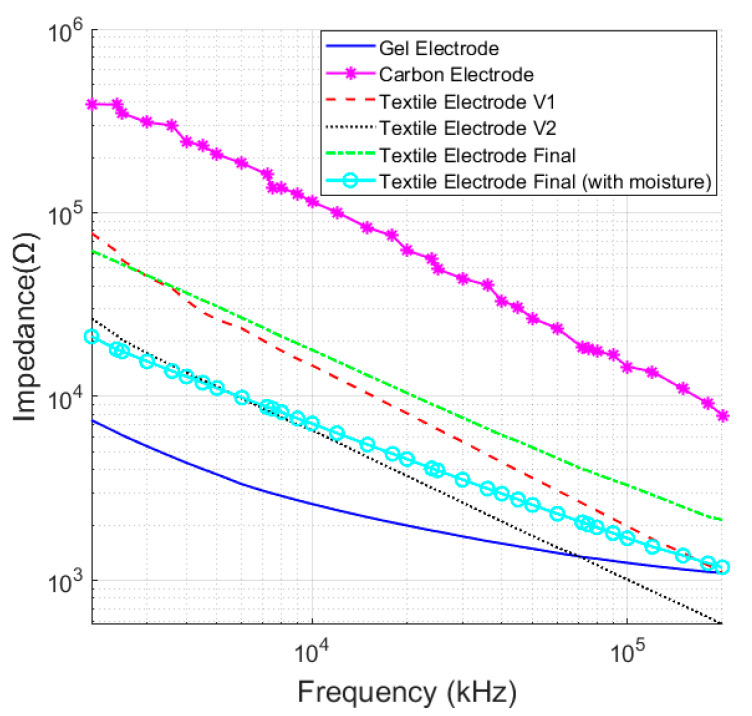
ETI measurement for different textile electrodes.

Two electrodes were positioned on the subject’s arm at a distance of 5 cm (center-to-center) for measurement. Figure 6 demonstrates the impedance variation for different versions of textile electrodes manufactured in this work. The ETI of a V1 textile electrode was higher than the gel electrode (under 10 kΩ for the whole frequency range) but lower than that of the carbon electrodes. However, for textile electrode V1, the curved nature of the electrode surface caused motion artifacts in the contact area, which introduced significant noise in the measured signal. V1 was further modified by using a more controlled padded cotton filler; however, there was not any major improvement noticed in the ETI measurement. Therefore, the filler material was changed from cotton to sponge to provide a better cushioning effect in version 2 (V2), as shown in Figure 4. The 4 cm × 2 cm conductive textile was wrapped around the sponge and fixed with medical-grade adhesive, which makes it easy to manufacture. This flat electrode provided stable contact with the subject’s skin. Figure 6 demonstrated significant improvement in ETI measurement compared to V1.

**Figure 7:**
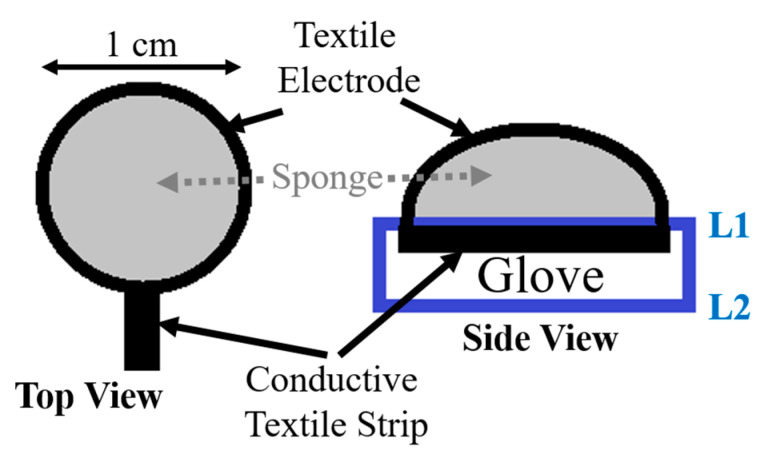
Schematic view of a manufactured final textile electrode.

However, the size of the electrode was still not appropriate for fitting within a glove, as proposed in this work. Therefore, a final 1 cm diameter circular electrode was manufactured (shown in Figure 4). The circular architecture also helped to avoid the generation of high current density at the sharp corners. Figure 7 shows the schematic view of the manufactured final version of the textile electrode.

**Figure 8:**
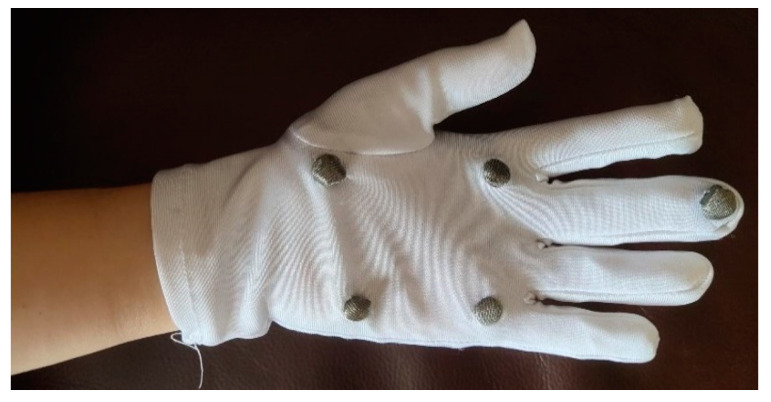
Manufactured glove with textile electrodes and a conductive strip.

A thin textile strip was sewn with the electrode to create a conductive path to connect with the RR measurement hardware. Furthermore, a soft two-layered glove was selected where a conductive textile was sewn in the first layer (L1) and another layer (L2) to create isolation between the conductive textile and the user’s skin. Figure 8 shows the manufactured glove with the textile electrodes attached to the palm of the glove where thin conductive strips were sewn to L1 of the glove and a medical grade connector button on the opposite side. The four electrodes inbuilt in the palm were used for bio-impedance measurement and a fifth electrode is kept in the middle finger for future use to measure ECG. The distance between the bio-impedance electrodes is approximately 5 cm (center to center). This was found to be optimal for children aged below 5 years. The optimal distance also considers the size of a glove worn by an adult female carer. The same electrode distance will, of course, fit in a larger glove (e.g., an adult male carer).

The ETI measurement of the final version of the textile electrode demonstrates an increase in impedance compared to its predecessor due to the significant reduction of the contact area. This is a necessary tradeoff accepted in this proposed system to accommodate the electrodes within a glove. However, it was observed that even a small amount of sweat on the skin could improve the contact and significantly reduce the impedance. To prove this, a small amount of moisture was used on the skin before conducting an ETI measurement. Figure 6 shows that it could bring the impedance of the final version of the electrode very close to the gel electrode.

**Figure 9:**
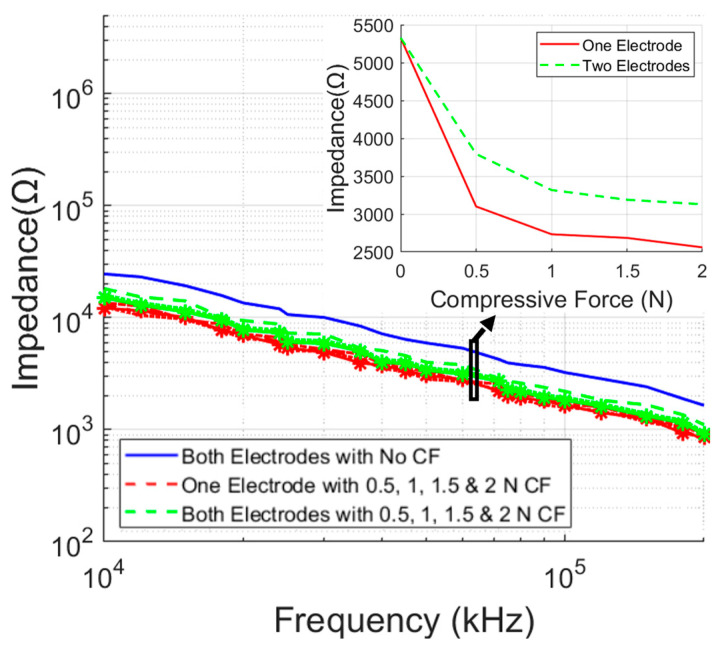
ETI measurement in final circular textile electrodes with varying compressive forces (without moisture applied).

Bioimpedance is a four-electrode measurement (described later), and the variation in ETI of individual electrodes should not affect the final results. However, due to manual, non-professional use of the gloves, different electrodes might experience a significantly different compressive force (CF). The effect of the CF on the ETI measurement was characterized using the final version of the electrodes by the method shown in Figure 5. It was tested with the varying CFs of 0.5, 1, 1.5, and 2 N, respectively, on one electrode first and then on both electrodes. In Figure 9, the red and green lines demonstrate the variation of the impedance due to different CFs on one or two electrodes and are compared when there was no CF present. There was no significant variation in the ETI that might affect the bioimpedance value. Figure 9 (inset) also shows the nature of the impedance change due to the variation of the CF at the excitation frequency of 62.5 kHz (the stimulation frequency used in the proposed system). A maximum CF of 2 N was selected for this experiment since anything further will cause irritation or discomfort to the infant. A light touch of the glove or a small pressure will generate much less CF.

## 3. System Design

**Figure 10:**
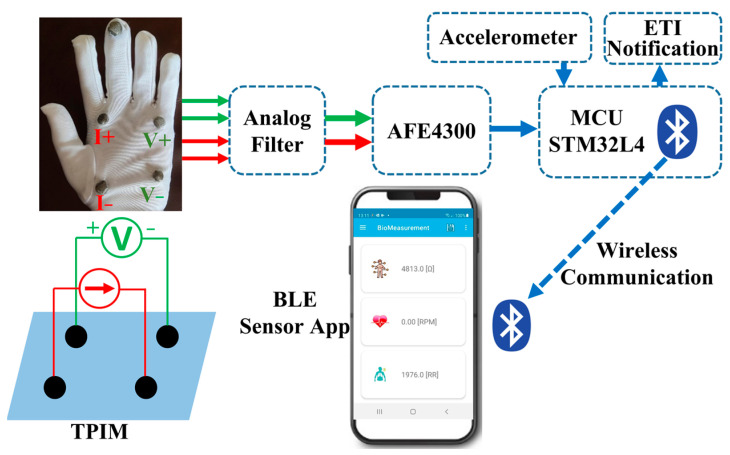
Proposed RR monitoring system block diagram.

Figure 10 shows the block view of the proposed respiratory rate monitoring system.

In conventional electrical impedance measurements, a bipolar configuration is used where the potential is developed across two terminals in a network due to a specific driven current. However, this technique is not suitable for bioimpedance measurements as tissue–electrode contact impedances are included in the measurement [29]. Therefore, the tetrapolar impedance measurement (TPIM) technique is adopted where separate pairs of electrodes are used for current drive and potential measurement. TPIM has different arrangements depending on the position of the electrodes. The most popular is a linear form where the current-driving electrodes and the voltage-measuring electrodes are in the same line [30]. In this method, the measurement-sensitive area is located close to the straight line between the two measuring electrodes and decreases the sensitivity significantly in the direction perpendicular to the straight line of the electrodes. However, considering the lungs’ unequal electrical conductivity throughout a wide area, the linear arrangement is not a suitable candidate for the proposed system. Therefore, the square electrode arrangement is adapted in this work. Two current driving electrodes and two potential difference measurement electrodes are positioned opposite to each other and form a square area that covers the lung area of a child (Figure 10). In Figure 10, *I*+ and *I*− are the electrodes for current excitation at 62.5 kHz and an amplitude of 20 µA (peak). *V*+ and *V*− are for voltage measurement across the sensitive area. In the proposed system, the electrode functions (current/voltage/polarity) can all be changed in quick succession, if desired (e.g., to gain better accuracy).

**Figure 11:**
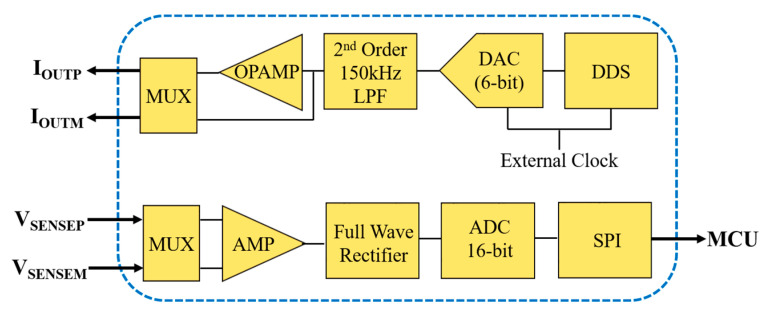
Body composition measurement block for bioimpedance measurement (full wave rectification method).

The input signal from the electrodes was filtered to remove the high frequency and dc elements before feeding it to the analog front end using a passive RC bandpass filter circuit with cutoff frequency of 0.15 Hz and 5 Hz. The analog front-end AFE4300 is an industrial standard integrated circuit (IC), which can be used for ECG and bioimpedance measurement. In this work, the full wave rectifier (FWR) mode of AFE4300 was used to compute the magnitude of the impedance using a single frequency. Figure 11 shows the simplified block diagram to measure bioimpedance using the full wave rectification method.

The drive current is generated by using direct digital frequency synthesis (DDS) technology with a 6-bit digital to analog converter (DAC) with a frequency conversion capability of up to 1 MHz and then filtered by 2nd order 150 kHz low pass filter (LPF). An opamp is used as a voltage-to-current converter and connected to the electrodes through a multiplexer (MUX). The potential difference generated across the skin due to the fixed frequency sinusoidal drive current can be measured by a differential amplifier connected through a MUX and converted to relevant dc voltage using an opamp-based full wave rectifier. The equivalent output of the measured impedance can be represented as:

(1) $$V_{IMP}=\frac{2}{T}\int_{\frac{T}{2}}^{}A\left|z\right|\sin\left(\omega_{0}t+\theta \right)dt=\frac{2A\left|z\right|}{\pi }$$

where *A* is the amplitude of the drive current and *z* is the measured impedance. The analog *V_IMP_* was then converted to a digital signal using a 16-bit analog to digital converter (ADC) in AFE4300 and transferred to the microcontroller unit (MCU) for further processing using a serial peripheral interface (SPI).

Along with bioimpedance, an accelerometer (LSM6DSLTR) is used to monitor the RR independently. We expect that lightly resting the palm (with the glove on) on the child’s chest will accurately monitor the breathing movement. The bioimpedance monitor could give us additional information on whether the glove is indeed touching the child’s chest by comparing the measured signal to a threshold. This is demonstrated as the ETI notification block in Figure 10. The user would be warned with a sound if the glove is not touching the chest appropriately for the accelerometer data to be valid.

**Figure 12:**
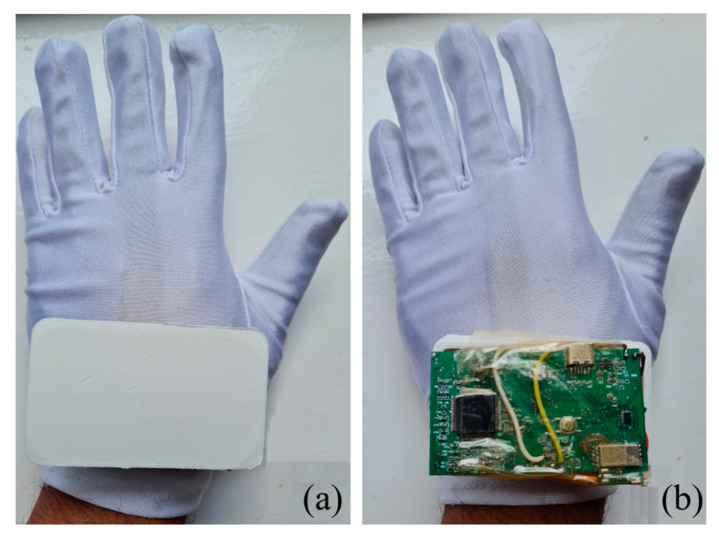
Fabricated RR monitoring system with the textile electrode glove. (**a**) System packaged into a 3D-printed case attached to the gloves. (**b**) The exposed PCB.

In this work, a low-power STM32L475E with Arm Cortex-M4E processor has been used as MCU which can read the data from both AFE4300 and accelerometer, process the data, and transmit the data to the mobile phone through the Bluetooth module SPBTLE-RFTR. Finally, the raw data from both sensors are observed using a custom ST-BLE Sensor mobile application. Figure 12a,b show the prototype printed circuit board (PCB) encased in a 3D printed box with a size of 75 cm × 46 cm × 24 cm attached to the back of the glove. The combined weight of the entire multimodal RR monitor PCB along with 3D-printed box and a rechargeable battery is close to 40 g. We do not expect that this will affect the accuracy of the measurement. The box also feels light when worn with the glove. An 1800 mAh rechargeable battery has been used in the proposed system, which can operate for approximately 20 h without recharging. Considering a conservative duration of 15 min (ON time) for each patient, the device could be used for 80 subjects before recharge.

**Figure 13:**
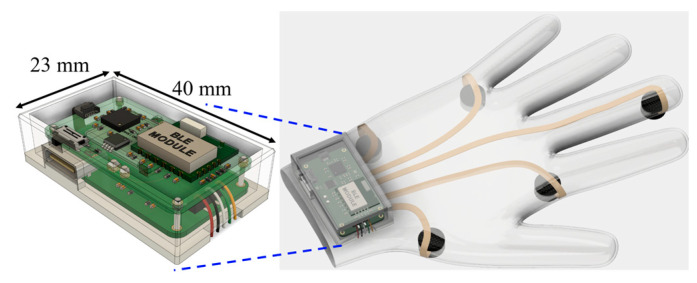
Schematic view of the optimised PCB attached to the proposed textile glove.

The volume of the prototype box is primarily driven by testing requirements. An optimized version of the manufactured PCB is in progress which can reduce the size significantly and fit in the textile glove even better compared to the current version. Figure 13 shows the schematic view of the optimized PCB with the proposed textile glove. Along with a rechargeable battery, the volume of the box can be 40 mm × 23 mm × 10 mm, which is almost half of the size of the current design. The weight of the new system will be approximately 15 g as a comparatively smaller battery will be used to accommodate the whole system in the package. With a 300 mAh battery the future system could operate for around 10 h without charging. 

## 4. Measurement

**Figure 14:**
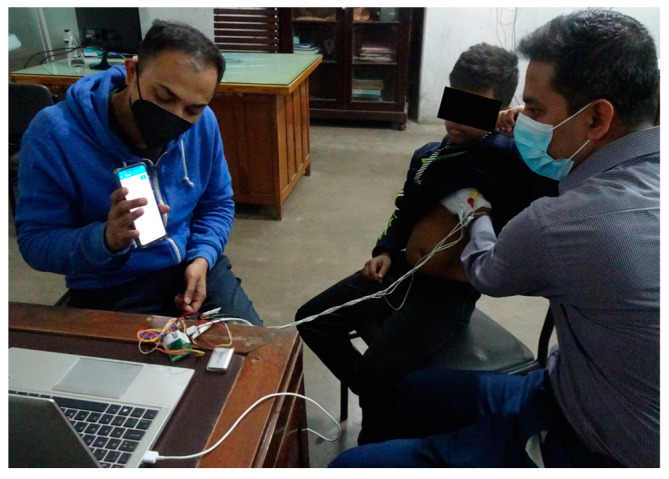
RR measurement on an 8-year-old volunteer at the Department of Biomedical Physics and Technology, University of Dhaka, Bangladesh.

The manufactured prototype was tested on healthy volunteers of different age groups. In Figure 14, the system was assessed on three children aged 3 to 10 years at the Department of Biomedical Physics and Technology, University of Dhaka, Bangladesh. To maintain the safety of the child, the electronics system (PCB and battery) was kept away, and the electrodes from the gloves were connected with medical-grade cables. The measurement was conducted in both the upper and lower chest (left and right sides) to select a proper measurement location. However, no significant difference was noticed in the measurement results for any part of the chest. Subjects did not report any discomfort when the glove was placed on them. Subjects were asked to breathe normally, and subsequently to take deep breaths and hold their breath. Younger subjects were instructed how to count RR and trained multiple times before conducting an RR measurement. Older subjects could easily self-report their own RR by counting each breath, which was instructed at the start of the experiment, and it could be further validated by observers conducting the experiment. The commonly used manual RR count was considered the gold standard [10,31,32].

The measurement setup took a few seconds before providing stable data on the mobile app. The setup time depends on the high-pass filter at the front of the signal chain. The data shown below are after the initial setup period. In a future implementation, this can be automated by thresholding the impedance value and discarding a few seconds of data.

The measured raw impedance signals consist of a biological and environmental artefact. They cannot be used directly in the final RR (or BPM) calculation. To facilitate RR detection, all signals are normalized after acquisition. Furthermore, a 2nd order low pass finite impulse response (FIR) filter with a cut-off frequency of 0.75 Hz and stopband attenuation of 65 dB has been used to eliminate high-frequency noise present in the measurements. A peak detection algorithm is then used to identify the peaks and count them. The measured bioimpedance result with textile electrodes is verified by accelerometer data collected at the same time. Accelerometer data are processed with the same algorithm as bioimpedance.

**Figure 15:**
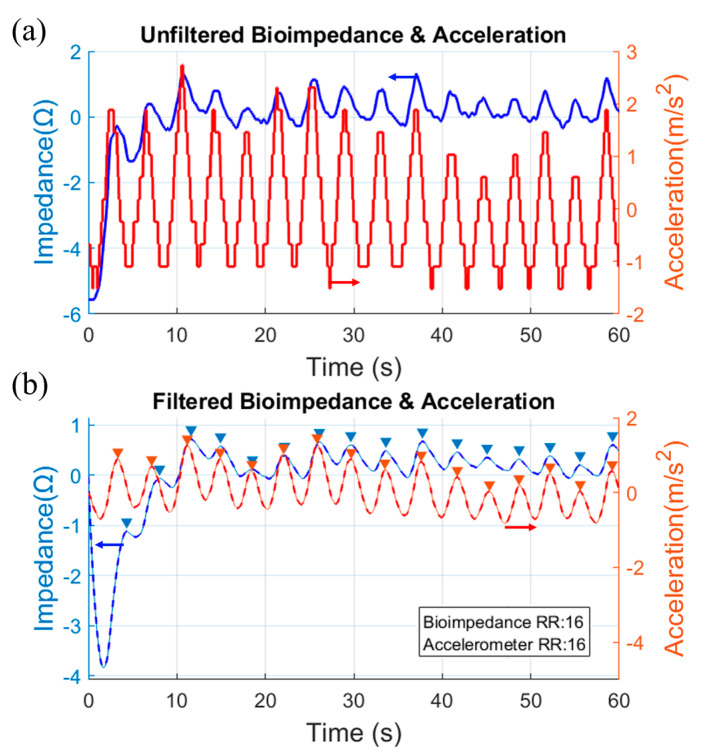
(**a**) Unfiltered and (**b**) filtered bioimpedance and acceleration data of a male volunteer of age 33.

Even if the electrode distance is not ideal for determining RR on an adult, we could easily achieve reasonably accurate results on a number of adult volunteers. Figure 15a,b show the unfiltered and filtered bioimpedance and acceleration plot of a male volunteer of age 33, respectively. The absolute magnitude of the impedance in the X-axis is unimportant and depends on a user-generated offset. This measurement was conducted using the glove demonstrated in Figure 12 where the PCB and the battery were directly connected to the electrodes. The filtered plot shows the picks to calculate the RR. The measured RR using the textile electrode is 16, which matches that from the acceleration data. It also matches the manually calculated breathing data.

**Figure 16:**
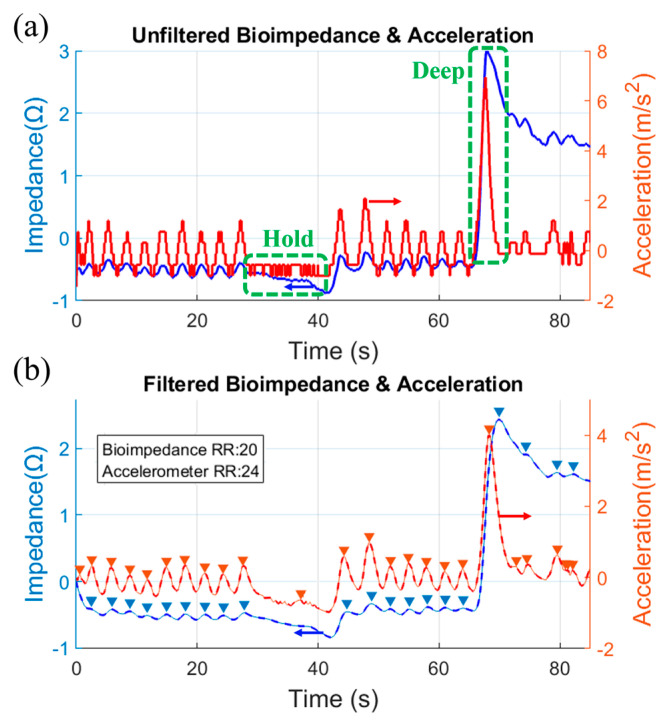
(**a**) Unfiltered and (**b**) filtered bioimpedance and acceleration data of a male volunteer of age 8.

In Figure 16a,b, the unfiltered and filtered bioimpedance and acceleration data from a child volunteer of age 8 are presented, respectively. It shows the data for 85 s, which cover different aspects of breathing. From 0 to 30 s, it shows normal breathing, 30 to 40 s for a breath-hold, and 65 to 70 s for a deep breath. The accelerometer and the bioimpedance data match closely in all cases. There is a variation of one RR count in the unfiltered acceleration data during the breath-holding time (it generated an extra pick). However, the filtered bioimpedance RR data match the manually counted data.

**Figure 17:**
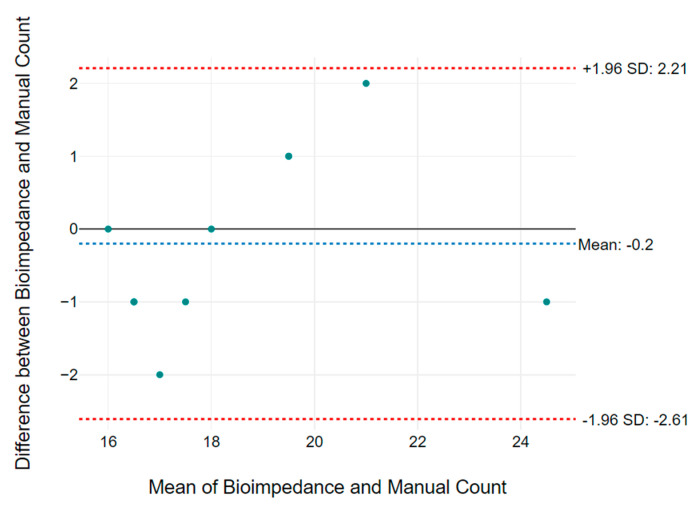
Bland Altman plot based on bioimpedance measured vs. manually counted RR values listed in Table 2.

**Figure 18:**
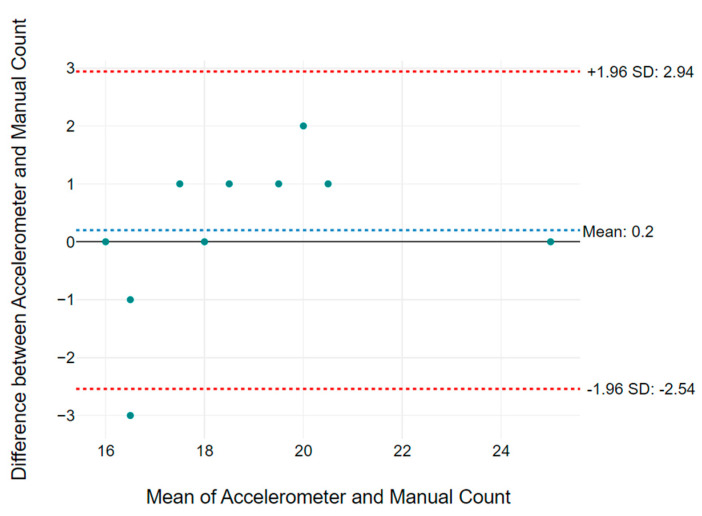
Bland Altman plot based on accelerometer vs. manually counted RR values listed in Table 2.

**Figure 19:**
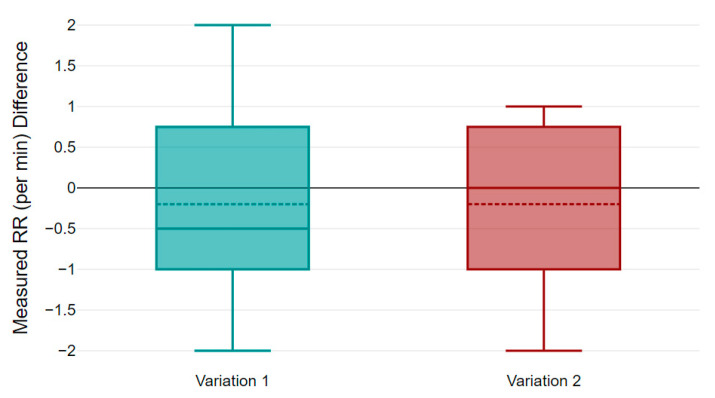
Boxplot of variation 1 and 2 of measured data.

Table 2 summarizes the RR results collected from volunteers of different age groups. It shows the filtered RR over 5 min of measurement calculated from unfiltered bioimpedance and acceleration data. The gold standard manually counted RR data to show the accuracy of the proposed system. The variation 1 (Var1) column shows the difference of RR between bioimpedance measurement using the textile electrodes and the manual count, while variation 2 (Var2) represents difference in RR between accelerometer and manual data. The data presented in Table 2 are averaged over 5 min and rounded to the nearest whole number. Figure 17 and Figure 18 show the Bland Altman plot for bioimpedance versus manually counted RR and accelerometer versus manually counted RR values, respectively, based on the data listed in Table 2. Figure 19 shows the boxplot of the variations 1 and 2 from Table 2. We can observe that the difference in measured RR in both cases lies within a box interval of 2 RR. The median and mean values of variation 1 are −0.5 and −0.2, respectively. Furthermore, median and mean values of variation 2 are 0 and −0.2, respectively. In both variations, the median and mean values are close to 0, which represents the accuracy of the proposed RR measurement system.

## 5. Conclusions

In this work, a novel portable multimodal RR monitoring system is presented. A custom-built glove with textile electrodes was used to measure RR using bioimpedance along with an accelerometer to confirm the accuracy of the data. Although separate instances of bioimpedance and accelerometer-based RR devices have been reported before, this is the first example of a multimodal RR measurement technique for early-age children. The glove and the dry electrodes make this a convenient and novel approach for diagnosing childhood pneumonia in infants in low-income countries. As demonstrated in medical literature, a distressed or crying child is still the most problematic issue in measuring accurate RR. Chest attachments or other unknown equipment are often a reason why infants become distressed. Hence, a lightly placed palm of a carer (wearing the proposed glove) on the child’s chest could be the most comforting action. In this work, the proposed device uses a natural, soothing posture to determine RR. The multimodal detection mechanism further enhances the usability of the device. The textile glove is easily washable by detaching the electronic box. This is a key and necessary feature for reusability in LMICs and reducing infection. The proposed RR monitoring system is tested on volunteers of different ages with good accuracy. Although designed for children, the same principle could be evaluated for use in adult age groups.

We demonstrate an accurate, easy-to-use, and affordable novel multimodal RR monitoring system which can support LMIC hospitals and healthcare centers to reduce the mortality rate among infants and young children from pneumonia. The technology could be adapted to also include ECG measurement in the future and warrants further development and evaluation.

## Figures and Tables

**Table 1:** Performance summary of different electrodes.

Electrodes	ETI	Comfort	Cost	Issues
Microneedle	Low	Low	High	Minor bleeding
Gel	Low	Moderate	Moderate	Skin irritation
Metal	High	Moderate	Low	Motion artifact
Foam	Moderate	High	Moderate	Complex manufacturing
Textile	Low	High	Low	Nothing major
Carbon	Low	High	Moderate	Complex manufacturing

**Table 2:** Summary of RR measurement.

Volunteer Age (Sex)	Average Manual Count (RR), per Min	Average Bioimpedance (RR), per Min	Average Accelerometer (RR), per Min	Var1, per Min	Var2, per Min
3 years (female)	25	24	25	−1	1
8 years (male)	18	18	19	0	−1
10 years (male)	20	22	21	+2	1
15 years (female)	17	16	16	−1	0
21 years (male)	19	20	21	1	−1
22 years (male)	17	16	18	−1	−2
22 years (female)	18	16	15	−2	1
27 years (male)	18	17	18	−1	−1
32 years (female)	19	20	20	+1	0
33 years (male)	16	16	16	0	0

## Data Availability

Not applicable.
